# Genetic parameters estimation and genome molecular marker identification for gestation length in pigs

**DOI:** 10.3389/fgene.2022.1046423

**Published:** 2023-01-05

**Authors:** Lijun Shi, Huihui Li, Lixian Wang

**Affiliations:** Institute of Animal Science, Chinese Academy of Agricultural Sciences, Beijing, China

**Keywords:** pig gestation length, genetic parameter estimation, GWAS, Fst, genetic molecular marker

## Abstract

Gestation length (GL) plays an important role in piglet maturation of major organs and development of body, while the genetic molecular markers of GL have not been extensively identified. In this study, according to the 5,662 effective records of 3,072 sows, the heritability and repeatability of GL were estimated through the dmuai of DMU Version 6.5.1 with a repeatability model, namely, $h^{2}$ = 0.1594 and ${re}^{2}$ = 0.2437. Among these sows, 906 individuals were genotyped with the GeneSeek Genomic Profiler (GGP) Porcine 50K Chip and imputed to the genome-wide level (9,212,179 SNPs) by the online software PHARP v1 for subsequent quality control and GWAS analyses. Further, the Fst was also performed to measure whether the actual frequency of genotypes in different GL phenotypes deviated from the theoretical proportion of genetic balance. We observed the highest degree of differentiation (average Fst value = 0.0376) in the group of 114 and 118 days, and identified a total of 1,002 SNPs strongly associated with GL. Through screening the genes located within a 500 kb distance on either side of the significant SNPs, we proposed 4,588 candidate genes. By the functional annotation, these candidates were found to be mainly involved in multicellular organism metabolism, early endosome, embryo implantation and development, and body and organ signaling pathway. Because of the simultaneous confirmation by GWAS and Fst analyses, there were 20 genes replied to be the most promising candidates including *HUNK*, *ARHGDIB*, *ERP27*, *RERG*, *NEDD9*, *TMEM170B*, *SCAF4*, *SOD1*, *TIAM1*, *ENSSSCG00000048838*, *ENSSSCG00000047227*, *EDN1*, *HIVEP1*, *ENSSSCG00000043944*, *LRATD1*, *ENSSSCG00000048577*, *ENSSSCG00000042932*, *ENSSSCG00000041405*, *ENSSSCG00000045589*, and *ADTRP*. This study provided effective molecular information for the genetic improvement of GL in pigs.

## Introduction

Gestation length (GL) is the interval (days) of sows from insemination to farrowing, which belongs to the reproduction traits. The GL of pigs is well documented as 3 months, 3 weeks, and 3 days (114 days). GL was influenced by the genotypes of piglets and sow (Rydhmer et al., 2008). Within the last few days of gestation, the piglet maturation of major organs and development of body mass occurs (Ullrey et al., 1965), herein, increasing GL might be utilized to improve piglet development and survivability. George Charbonneau, a veterinarian, talked about “Keeping Large Litters Alive” and shared the attached two charts at the Shakespeare Swine Seminar (https://www.nationalhogfarmer.com/business/gestation-length-and-inducing-what-do-we-know), showing that sows allowed to farrow at day 118 had pigs born at an average weight of 3.7 pounds versus day 113 at 2.9 pounds (Supplementary Figure S1A) and indicating that earlier a sow farrows the lower the level of Immunoglobulin G colostrum in the milk for the pigs (Supplementary Figure S1B). The gain of GL was negatively correlated with litter size, while was positively correlated with the average pig weight (Omtvedt et al., 1965; Leigh, 1981). Selection for prolonged gestation might probably improve piglet survival after birth and piglet growth, while it might result in more stillbirths (Rydhmer et al., 2008). Hence, it is very important to understand the genetic mechanism of pregnancy days in pigs.

GL is not measurable until sexual maturity, and traditional breeding technologies are limited. Genomic selection improving the efficiency and accuracy of breeding is a form of marker-assisted selection in which genetic markers covering the entire genome are used (Yang et al., 2020). Some of the early reports proposed quantitative trait loci (QTL) and novel genetic variants associated with pig GL, while the limited number of DNA markers and F2 populations with large linkage disequilibrium blocks resulted in large genomic regions associated with GL (Wilkie et al., 1999; Chen et al., 2010). There were some potential QTLs, variants, and genes associated with GL of swine (Onteru et al., 2012; Hidalgo et al., 2016; Wang et al., 2018; See et al., 2019), while the relevant research was still lacking.

In this study, we obtained 5,662 valid GL records from 3,097 pigs, and adopted a strict repeatability model to estimate the heritability of GL. To identify the genetic SNPs and genes associated with GL, we conducted the GWAS analyses according to the GGP Porcine 50K Chip, imputed SNPs and performed the Fst analysis. Further, we carried out the functional enrichments to annotate these candidate genes. This research will provide valuable genetic resources for the future understanding of GL regulation mechanisms and breeding work.

## Materials and methods

### Animals and phenotype collection

A total of 3,072 pigs were used in the present study, and they were from two farms in Liaoning and Shanxi Provinces, China. The GL, breed, farm, parity, and total litter birth weight were collected for these sows, and 5,662 records were shown in Supplementary Table S1. The pedigrees of these sows were traced to four generations with 5,786 individuals to be used to estimate the genetic parameters of GL.

### Genetic parameter estimation

By the dmuai of DMU Version 6.5.1, we estimated the variance and covariance components of GL based on a repeatability model, which was described as follows:

$$y=Xb+Z_{a}a+Z_{p}p+e$$

in which, y was the vector of GL phenotype; b was the vector of fixed effects including farm (two farms), breed (three variables: Yorkshire, Landrace, and Duroc pigs), pairty (four variables: 1, 2, 3, and 4–8 parity), and total litter birth weight (31 variables: 1–29, 32, and 33 kg); X was the design matrix associated b with y ; p was the vector of random permanent environmental effects; a was the vector of additive genetic effects; $Z_{p}$ and $Z_{a}$ referred to the corresponding residual effects; and e was the vector of random residual effects.

Further, we calculated the heritability and repeatability of GL by the following formulas:

$$h^{2}=\frac{\sigma_{a}^{2}}{\sigma_{a}^{2}+\sigma_{p}^{2}+\sigma_{e}^{2}}\;and\;{re}^{2}=\frac{\sigma_{a}^{2}+\sigma_{p}^{2}}{\sigma_{a}^{2}+\sigma_{p}^{2}+\sigma_{e}^{2}};$$

where $h^{2}$ and ${re}^{2}$ were the heritability and repeatability, respectively. $\sigma_{a}^{2}$ was the additive genetic variance, $\sigma_{p}^{2}$ was the permanent environmental effect variance, and $\sigma_{e}^{2}$ was the residual effect variance.

### Quality control of genotype data

Of all 3,072 sows with GL phenotypes, 906 individuals were used for GWAS. The ear samples of these 906 sows were collected and the DNA was isolated from them by a commercially available kit (Q1Aamp DNA Mini Kit, QIAGEN, Germany). Then these pigs were genotyped with the GeneSeek Genomic Profiler (GGP) Porcine 50K Chip (Illumina, San Diego, CA, United States), which contained 50,697 SNPs. With the PLINK, we performed the quality control by removing the SNPs with Minor Allele Frequencies (MAF) < 0.05, call rate <95%, and a deviation from Hardy-Weinberg equilibrium (HWE, *p* < 0.000001). Finally, we obtained 34,609 SNPs and 906 animals for GWAS, in which, all the individuals had a high SNP detection rate (>90%). All SNP positions were annotated according to the pig genome assembly Sscrofa 11.1.

### Genotype imputation

To improve the identification of QTL, the whole-genome sequence data is expected to use (van den Berg et al., 2019), while sequencing thousands of individuals is expensive. In this study, we conducted the imputation for the GGP Porcine 50K Chip after quality control according to the online software PHARP v1 (http://alphaindex.zju.edu.cn/PHARP/index.php), which included 2,012 haplotypes, 34 million SNPs of autosomes, and 71 pig breeds (Wang et al., 2022). After imputation, we carried out the quality control (*R* ^2^ ≥ 0.8 and MAF = 0.05), and obtained 9,212,179 SNPs, in which, the average *R* ^2^ was 0.9248. Further, we conducted the quality control and GWAS according to the filter and model used in the front GGP Porcine 50K Chip, where 9,043,409 SNPs and 906 individuals were utilized.

### Genome-wide association study

We conducted the GWAS by GCTA software with the model as follows:

y=μ+bX+G+F+P+m×M+e

where y is a vector of phenotype values for all individuals; b is the average effects of gene substitution of a particular SNP; X is a vector of the SNP genotype; G is the genomic relationship matrix created by all SNPs; F and P were the farm (two factors) and parity (four factors) fixed effects, respectively; M is the fixed effects of total litter birth weight; m is the regression coefficient of M and e is the random residual.

The results of GWAS were shown by Manhattan and quantile-quantile (Q-Q) plots. A significant SNP was determined if the raw *p*-value <0.05/n, in which, n was the number of SNPs. Further, the threshold of suggestive significant association was calculated according to 1/n.

### F-statistics analysis

In population genetics, Fst is an index to measure whether the actual frequency of genotypes in a population deviates from the theoretical proportion of genetic balance. In recent years, the number of litter sizes has increased, and the GL has been obvious for a long. To evaluate the degree of genetic differentiation of individuals with the different GL, Fst was carried out through the vcftools software. We calculated the Fst value of each SNP between GL 114 days and 115/116/117/118 days based on the following formulas:

$$F_{ST}=\frac{H_{T}-H_{S}}{H_{T}},$$

$$H_{T}=2\times \left(1-\frac{P_{E}+P_{F}}{2}\right)\times \frac{P_{E}+P_{F}}{2},$$

and

$$H_{S}=\frac{2\times P_{E}\times \left(1-P_{E}\right)+2\times P_{F}\times \left(1-P_{F}\right)}{2};$$

In which, $P_{E}$ and $P_{F}$ are the allele frequencies of individuals with GL 114 days and 115/116/117/118 days, respectively.

The SNPs involved in the top 1% (Fst values) were selected as the core SNPs associated with the GL trait.

### Candidate genes and functional annotation

By BioMart of Ensembl database, the candidate genes within 500 kb of significant SNPs were screened based on the pig reference genome (Sscrofa11.1). Further, we investigated the functions of these genes through the KOBAS (http://kobas.cbi.pku.edu.cn/kobas3/genelist/), and the significance threshold was corrected *p*-value < 0.05.

## Results

### Phenotypes and genetic parameters of gestation length

**FIGURE 1:**
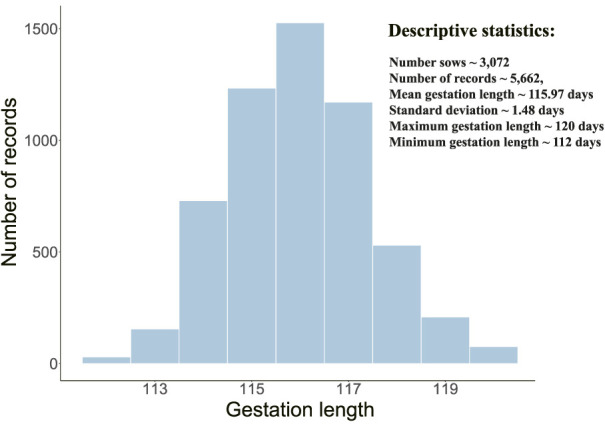
GL phenotype distribution and descriptive statistics.

GL phenotype distribution and descriptive statistics were shown in Figure 1. The average GL was 115.97 days in this population, and the maximum and minimum values were 120 and 112, respectively. By estimation, the additive genetic variance V(a), permanent environmental effect variance V(p), and residual effect variance V(e) were detected, namely, 0.3014, 0.1594, and 1.4299, respectively. Correspondingly, the heritability and repeatability were calculated: $h^{2}$ = 0.1594 and ${re}^{2}$ = 0.2437.

### Identification of significant SNPs associated with GL by genome-wide association study

**FIGURE 2:**
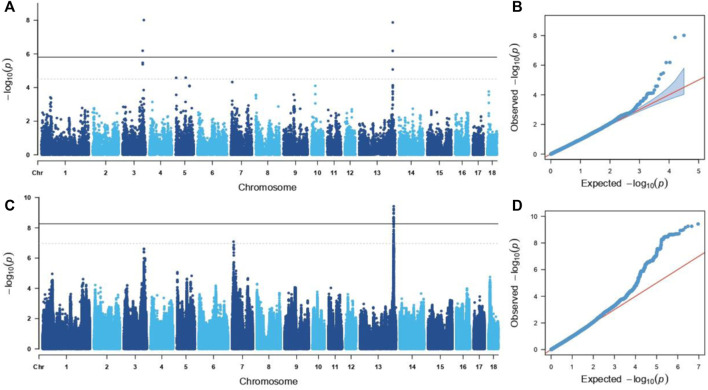
Manhanttan and qq plots of GWAS for GL. The solid and dotted lines indicated significant and suggestive significant thresholds, respectively. **(A,B)**. GWAS results based on the GGP Porcine 50K Chip. **(C,D)**. GWAS results were based on the SNPs imputed.

Through the GWAS based on the GGP Porcine 50K Chip, four genome-wide significant SNPs were identified (*p* < 1.44E-06 (0.05/34,609), Table 1 and Figures 2A,B), including WU_10.2_8_32052217 and WU_10.2_3_124292040 located on (*Sus scrofa* Chromosome (SSC) 3, and ALGA0073634 and WU_10.2_13_204954425 located on SSC13). According to the suggestive significant threshold *p* < 2.89E-05 (1/34,609), additional five SNPs were detected (Table 1 and Figures 2A,B), namely, WU_10.2_3_124371187 and WU_10.2_3_124478097 located on SSC3, H3GA0016351 and WU_10.2_5_111419379 located on SSC5, and CASI0009756 located on SSC13.

**TABLE 1:** The significant SNPs identified by GWAS analysis of GGP Porcine 50K Chip.

SNP	Location	SNP effect	*p*-value	Candidate gene
WU_10.2_8_32052217	chr3:123152070	−0.359903	9.77E-09	ENSSSCG00000043944
				*LRATD1*
				ENSSSCG00000048577
				ENSSSCG00000042932
				ENSSSCG00000041405
				ENSSSCG00000045589
ALGA0073634	chr13:194840009	0.390875	1.35E-08	ENSSSCG00000039409
				*KRTAP11-1*
				*SCAF4*
				*SOD1*
				*TIAM1*
WU_10.2_3_124292040	chr3:116471054	0.452164	6.51E-07	ENSSSCG00000047227
				ENSSSCG00000048838
WU_10.2_13_204954425	chr13:194555673	0.326668	6.64E-07	ENSSSCG00000039409
				*KRTAP11-1*
				*TIAM1*
WU_10.2_3_124371187	chr3:116704665	0.368326	3.37E-06	ENSSSCG00000048838
WU_10.2_3_124478097	chr3:116746742	0.362195	4.17E-06	ENSSSCG00000048838
CASI0009756	chr13:194739193	0.287076	8.45E-06	ENSSSCG00000039409
				*KRTAP11-1*
				*TIAM1*
H3GA0016351	chr5:57889765	0.321036	2.59E-05	*MGP*
				*SMCO3*
				*H2AJ*
				*ARHGDIB*
				*ERP27*
				*ART4*
				ENSSSCG00000000611
				*RERG*
				*PLBD1*
				*ATF7IP*
WU_10.2_5_111419379	chr5:2378170	0.264446	2.65E-05	ENSSSCG00000034275
				*CERK*
				*TBC1D22A*
				*GRAMD4*

### Genome-wide association study based on the imputation genotype data

Further, the genotype was imputed by PHARP v1 to be used for a GWAS, and a total of 53 genome-wide significant SNPs [*p* < 5.52E-09 (0.05/9,043,409)] on pig chromosomes SSC13 were identified (Figures 2C,D; Table 2), including SNP ALGA0073634. Based on the suggestive significant threshold *p* < 1.11E-07 (1/9,043,409), 112 SNPs were detected to be associated with GL, containing 111 located on SSC13 and one located on SSC7 (Figures 2C,D; Table 2).

**TABLE 2:** The significant SNPs identified by GWAS analysis of imputed SNPs.

SNP	Location	*p*-value (<)	Candidate gene
chr13:194845972:A:G, chr13:194845916:G:C, chr13:194845971:C:T, chr13:194845415:T:C, chr13:194845412:T:G, chr13:194844055:T:C, chr13:194844073:G:A, chr13:194839531:G:C, chr13:194839600:T:C, chr13:194839604:G:A, chr13:194840157:T:C, chr13:194840386:C:T, chr13:194842844:G:C, chr13:194843153:G:A, chr13:194843293:T:C, chr13:194844138:T:C, chr13:194844319:T:C, chr13:194844405:T:C, chr13:194844842:T:C, chr13:194844898:T:C, chr13:194845221:G:A, chr13:194845341:T:C, chr13:194841615:T:C, chr13:194844736:T:C, chr13:194844756:G:A, chr13:194842856:A:C, chr13:194843152:C:T, chr13:194843190:C:T, chr13:194844293:A:G, chr13:194844298:G:A, chr13:194844320:T:C, chr13:194844393:C:T, chr13:194844679:A:G, chr13:194845225:C:G, chr13:194840587:G:T, chr13:194840628:G:C, chr13:194840706:C:A, chr13:194840748:C:G, chr13:194841022:G:A, chr13:194844244:T:C, chr13:194844245:G:A, chr13:194839529:G:A, chr13:194839588:G:A, chr13:194839971:G:A, ALGA0073634, chr13:194840084:T:C, chr13:194840099:G:A, chr13:194840780:C:T, chr13:194846836:A:T, chr13:194846842:C:A, chr13:194846853:T:A, chr13:194841212:C:T, chr13:194841762:C:G, chr13:194840206:T:C, chr13:194840737:T:G, chr13:194841443:T:C, chr13:194841499:C:T	chr13:194845972, chr13:194845916, chr13:194845971, chr13:194845415, chr13:194845412, chr13:194844055, chr13:194844073, chr13:194839531, chr13:194839600, chr13:194839604, chr13:194840157, chr13:194840386, chr13:194842844, chr13:194843153, chr13:194843293, chr13:194844138, chr13:194844319, chr13:194844405, chr13:194844842, chr13:194844898, chr13:194845221, chr13:194845341, chr13:194841615, chr13:194844736, chr13:194844756, chr13:194842856, chr13:194843152, chr13:194843190, chr13:194844293, chr13:194844298, chr13:194844320, chr13:194844393, chr13:194844679, chr13:194845225, chr13:194840587, chr13:194840628, chr13:194840706, chr13:194840748, chr13:194841022, chr13:194844244, chr13:194844245, chr13:194839529, chr13:194839588, chr13:194839971, chr13:194840009, chr13:194840084, chr13:194840099, chr13:194840780, chr13:194846836, chr13:194846842, chr13:194846853, chr13:194841212, chr13:194841762, chr13:194840206, chr13:194840737, chr13:194841443, chr13:194841499	3.42E-08	*ENSSSCG00000039409, KRTAP11-1, SCAF4, SOD1, TIAM1*
chr13:194578689:C:T, chr13:194572528:G:A, chr13:194569322:G:A, chr13:194570051:A:G, chr13:194572470:A:T, chr13:194567558:G:C, chr13:194569012:C:T, chr13:194569022:G:A, chr13:194569063:A:G, chr13:194569139:T:C, chr13:194569144:T:C, chr13:194569161:C:T, chr13:194569168:G:A, chr13:194569383:C:T, chr13:194569417:A:G, chr13:194569554:T:C, chr13:194569620:A:G, chr13:194569726:A:T, chr13:194569986:T:A, chr13:194570090:C:T, chr13:194570108:A:G, chr13:194570252:T:G, chr13:194570272:T:A, chr13:194573199:G:A, chr13:194573657:A:C, chr13:194574481:T:C, chr13:194575322:G:A, chr13:194576218:G:A, chr13:194575750:T:G, chr13:194569070:A:G, chr13:194572195:G:C, chr13:194572541:C:A, chr13:194572070:C:A, chr13:194577916:G:A, chr13:194559261:C:T, chr13:194556306:G:A, chr13:194557487:T:A, chr13:194561479:A:G, WU_10.2_13_204954425, chr13:194555839:C:T, chr13:194556910:G:T, chr13:194557274:T:C, chr13:194557379:G:A, chr13:194557581:A:T, chr13:194557936:C:T, chr13:194557963:T:C, chr13:194558256:A:G, chr13:194562758:A:G	chr13:194578689, chr13:194572528, chr13:194569322, chr13:194570051, chr13:194572470, chr13:194567558, chr13:194569012, chr13:194569022, chr13:194569063, chr13:194569139, chr13:194569144, chr13:194569161, chr13:194569168, chr13:194569383, chr13:194569417, chr13:194569554, chr13:194569620, chr13:194569726, chr13:194569986, chr13:194570090, chr13:194570108, chr13:194570252, chr13:194570272, chr13:194573199, chr13:194573657, chr13:194574481, chr13:194575322, chr13:194576218, chr13:194575750, chr13:194569070, chr13:194572195, chr13:194572541, chr13:194572070, chr13:194577916, chr13:194577182, chr13:194559261, chr13:194556306, chr13:194557487, chr13:194561479, chr13:194555673, chr13:194555839, chr13:194556910, chr13:194557274, chr13:194557379, chr13:194557581, chr13:194557936, chr13:194557963, chr13:194558256, chr13:194562758	1.03E-07	*ENSSSCG00000039409, KRTAP11-1, TIAM1*
chr13:195122765:C:T, chr13:195099333:A:G, chr13:195099213:G:A, chr13:195099365:T:G	chr13:195122765, chr13:195099333, chr13:195099213, chr13:195099365	1.04E-07	*SCAF4, SOD1, HUNK, TIAM1*
chr13:194561368:T:C	chr13:194561368	1.10E-07	
chr7:8533647:A:T	chr7:8533647	8.22E-08	*EDN1, ENSSSCG00000041448, NEDD9, TMEM170B, ADTRP, HIVEP1*

### Detection of score SNPs associated with gestation length by F-statistics analysis

**FIGURE 3:**
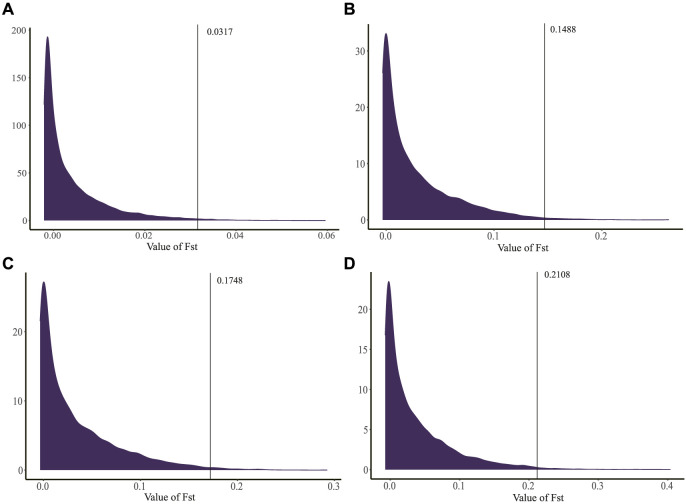
Distribution of Fst density on chromosome. **(A)** Analysis between 114 and 115 days. **(B)** Analysis between 114 and 116 days. **(C)** Analysis between 114 and 117 days. **(D)** Analysis between 114 and 118 days. The black lines indicated 320 SNPs in the top 1% (FST > 0.0317, 0.1489, 0.1748 and 0.2108 for 114 and 115/116/117/118 days, respectively).

GLs of 114, 115, 116, 117, and 118 days were involved in 309, 263, 116, 117, and 49 individuals, respectively. Based on the GGP Porcine 50K Chip data, we calculated the Fst values of GL between 114 days and 115/116/117/118 days for each SNP, and the FST density distribution maps were constructed (Figure 3). The average Fst values of the four groups were 0.0041, 0.0279, 0.0341, and 0.0376, indicating that there was a differentiation between individuals with GL of 114 days and 115/116/117/118 days. In addition, the group of 114 and 118 days had the highest degree of differentiation than other groups.

**FIGURE 4:**
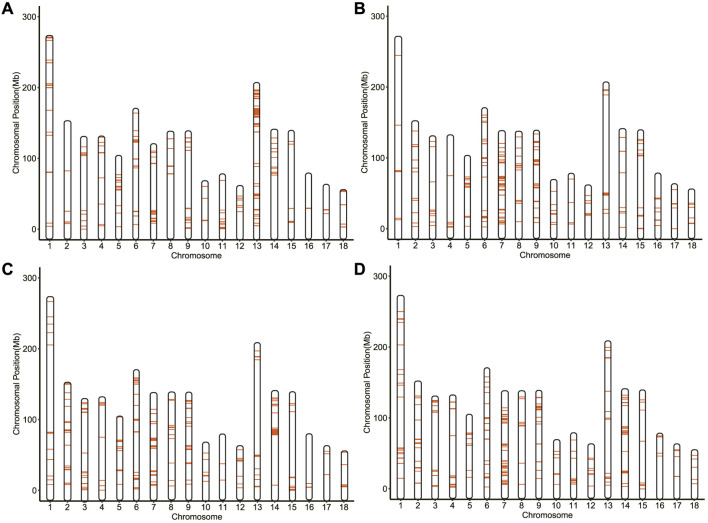
The maps of 320 selection signatures on pig chromosome. **(A)** Analysis between 114 and 115 days. **(B)** Analysis between 114 and 116 days. **(C)** Analysis between 114 and 117 days. **(D)** Analysis between 114 and 118 days.

For each group, 320 SNPs in the top 1% (FST > 0.0317, 0.1489, 0.1748, and 0.2108 for 114 and 115/116/117/118 days, respectively) were the core SNPs in the region where the signal occurred (Figure 3), a total of 885 core SNPs were identified (Supplementary Table S2). Out of them, SSC13 had the largest number of core SNPs with 119, followed by SSC7 (116 core SNPs) and SSC9 (89 core SNPs). The highest FST value (FST = 0.4035) was located on SSC1. According to the chromosomal location and FST value of the selection signal, the distribution of 885 core SNPs for each group was marked in Figure 4, which showed the differences in the distribution of selection signals related to GL across the genome. Combined with GWAS results, four SNPs, WU_10.2_8_32052217, WU_10.2_3_124292040, WU_10.2_3_124371187 and WU_10.2_3_124478097 located on SSC3, were co-identified by GWAS and Fst analysis.

### Screening candidate genes associated with gestation length and functional annotation

The genes located within a 500 kb distance on either side of the significant SNPs were considered candidates for GL. In total, 4,588 candidate genes were detected, of which, 27 were identified by GWAS of GGP Porcine 50K Chip data (Table 1), 12 were identified by GWAS of imputation SNPs (Table 2), and 4,574 were screened by core SNPs of Fst analysis (Supplementary Table S2). There were 20 genes simultaneously confirmed by GWAS and Fst analysis, namely *HUNK*, *ARHGDIB*, *ERP27*, *RERG*, *NEDD9*, *TMEM170B*, *SCAF4*, *SOD1*, *TIAM1*, *ENSSSCG00000048838*, *ENSSSCG00000047227*, *EDN1*, *HIVEP1*, *ENSSSCG00000043944*, *LRATD1*, *ENSSSCG00000048577*, *ENSSSCG00000042932*, *ENSSSCG00000041405*, *ENSSSCG00000045589*, and *ADTRP*.

To research the functions of candidate genes, we performed functional annotation by KOBAS. 12 genes from GWAS of GGP Porcine 50K Chip data were significantly involved in four KEGG pathways and 84 GO terms, including protein and lipid metabolism, regulation of multicellular organism growth, embryo implantation, and ovarian follicle development (Supplementary Table S3). Seven candidates from GWAS of imputation SNPs were strongly involved in 11 KEGG and 109 GO enrichments, which were mainly associated with body and organ signaling pathways, cellular metabolism, regulation of neuron apoptotic process, and heart development (Supplementary Table S3). 2,518 genes from Fst results heavily participated in 24 KEGG and 128 GO enrichments, containing cell differentiation, early endosome, and carbohydrate metabolic process etc. (Supplementary Table S3).

## Discussion

GL is closely related to sow reproductive traits, such as litter size, herein deciphering the genetic mechanism of GL has important implications for genomic selective breeding. In the present study, we obtained the GL trait of pigs from a separate farm and estimated the heritability based on the 5,662 records of 3,072 sows. A total of 906 individuals were genotyped by 50K SNP Chip, and used for the GWAS by GCTA. Also, the genotypes were imputed to 9,212,179 SNPs for a further GWAS. In addition, we conducted the Fst analysis to detect more genetic markers for the GL trait. For these SNPs and candidate genes, we searched their enrichment annotation to provide preliminary functional analysis. While, the biological mechanism of these genetic associations between the SNPs or genes with GL still needs to be verified in future research.

The heritability of GL was estimated by a repeatability model with the pooling data of three breeds and was shown with $h^{2}$ = 0.1594. Previous research reported that GL is a moderate heritability trait and its heritability is approximately 0.25–0.29 in a Dutch Landrace population (Hanenberg et al., 2001), 0.29 for Landrace pigs and 0.34 for Yorkshire pigs (Ogawa et al., 2019), around 0.2 in Landrace pigs (Crump et al., 1997), 0.16 in the populations of Landrace, Yorkshire and their crosses (Imboonte and Kuhaaudomlarp, 2012), 0.18 for Hungarian Landrace and 0.26 for Hungarian Large White pigs (Nagy et al., 2009), and 0.21 for two breeds of Large White and Landrace sows (Hollema et al., 2020). Comparing the heritability of GL reported, the heritability of GL estimated in this study was a bit low, which might be due to the different population structures. Overall, it is consistent with the previously reported results, and these results indicated that GL was a heritable trait, and provides the basis for improving phenotypes through genomic selection at a later stage.

By the GWAS, nine and 112 SNPs significantly associated with GL were identified by GGP Porcine 50K Chip and imputed SNPs, respectively, and 34 candidate genes were screened. Among them, five genes located on SSC13 were identified at the same time by two association analyses. ENSSSCG00000039409 was a novel gene, and its function study was insufficient until now. *KRTAP11-1* was a hair keratin-associated protein, as a possible crucial element for the physical properties of hair shafts, and was related to the embryonic hair development of Foxn1nu mice (Fujimoto et al., 2014). *SCAF4* encoded the member of the serine/arginine-rich splicing factor family, which was an antiterminator during transcription (Gregersen et al., 2019). *SCAF4* was important for correct usage of polyA sites for mRNA termination, and might be associated with the variable neurodevelopmental disorder (Fliedner et al., 2020). *SOD1* was involved in fetal loss of women (Zhang et al., 2019) and muscle development (Abati et al., 2020). *TIAM1* was part of the AMPK-Tiam1-Rac1 signaling pathway, which mediates contraction-stimulated glucose uptake in skeletal muscle cells and tissues (Yue et al., 2021). The lack of the Tiam1/Rac1 signal would contribute to the inhibition of embryo implantation in mice (Ma et al., 2015).

Fst is a measure of population divergence, and it is widespread to quantify the genetic distance between populations and to assess differentiation at individual SNPs by Fst (Bhatia et al., 2013). In this study, we observed the Fst values between 114 days and 115/116/117/118 days for each SNP, and the degree of differentiation was gradually increased from the groups of 114 and 115 days to 114 and 118 days, which meant the highest divergence between GL of 114 and 118 days. Combined with GWAS results, 20 genes were simultaneously identified through GWAS and Fst analysis, out of them, *SCAF4*, *SOD1*, and *TIAM1* were detected in two GWASs, and *ENSSSCG00000048838*, *ENSSSCG00000047227*, *ENSSSCG00000043944*, *ENSSSCG00000048577*, *ENSSSCG00000042932*, *ENSSSCG00000041405*, and *ENSSSCG00000045589* were the novel genes. *HUNK* was involved in transferase activity, transfer phosphorus-containing groups, and protein tyrosine kinase activity, and was thought to play a role in breast cancer metastasis (Dilday et al., 2020; Williams et al., 2020). *ARHGDIB*, *RERG*, and *TMEM170B* were also related to breast cancer (Li et al., 2018; Huang et al., 2020; Wang et al., 2020), implying they might participate in the development of mammary glands. *ERP27* was a redox-inactive member of the PDI family, and enhanced receptor stability and downstream signaling maintain the stability of the organism (Kober et al., 2013). *NEDD9* was found to regulate the developing embryonic nervous system (Knutson and Clagett-Dame, 2015), and *HIVEP1* was involved in the obesity of newborns (Rizzo et al., 2020). *ADTRP* played a critical role in vascular development and vessel integrity and function (Lupu et al., 2021), and also specifies primitive myelopoiesis and definitive hematopoiesis (Luo et al., 2020). LRATD1 was the substrate protein of NMT1/2 (Su et al., 2021) and involved in cell morphogenesis and cell motility. *EDN1* was linked to the local hemodynamic response of uteroplacental, and was a key player in the pathophysiology of hypoxia-induced fetal growth restriction (Thaete et al., 2007), and its signaling was crucial for the formation of the embryonic pharyngeal arches and their skeletal derivatives (Sasaki et al., 2013).

## Conclusion

In the present study, the heritability 0.1594 of GL was shown, and a total of 1,002 SNPs were detected to impact GL phenotype by GWAS analyses of GGP Porcine 50K Chip and imputed SNPs, and Fst analysis. Further, 4,588 genes were considered candidates for GL, and their functions were confirmed, in which, 20 genes simultaneously confirmed by GWAS and Fst analysis were the most suggestive candidates.

## Data Availability

All raw data generated in this study were provided in this study and public repository (https://figshare.com/s/edda38a1c99aa7ab7ae0).

## References

[B1] Abati E. Bresolin N. Comi G. Corti S. (2020). Silence superoxide dismutase 1 (SOD1): A promising therapeutic target for amyotrophic lateral sclerosis (ALS). Expert Opin. Ther. Targets 24 (4), 295–310. 10.1080/14728222.2020.1738390 32125907

[B2] Bhatia G. Patterson N. Sankararaman S. Price A. L. (2013). Estimating and interpreting FST: The impact of rare variants. Genome Res. 23 (9), 1514–1521. 10.1101/gr.154831.113 23861382 PMC3759727

[B3] Chen C. Y. Guo Y. M. Zhang Z. Y. Ren J. Huang L. S. (2010). A whole genome scan to detect quantitative trait loci for gestation length and sow maternal ability related traits in a White Duroc × Erhualian F2 resource population. Animal 4 (6), 861–866. 10.1017/S1751731110000169 22444258

[B4] Crump R. E. Haley C. S. Thompson R. Mercer J. (1997). Individual animal model estimates of genetic parameters for reproduction traits of Landrace pigs performance tested in a commercial nucleus herd. Anim. Sci. 65, 285–290. 10.1017/s1357729800016593

[B5] Dilday T. Ramos N. Yeh E. (2020). HUNK signaling in metastatic breast cancer. Oncoscience 7 (5-6), 30–33. 10.18632/oncoscience.504 32676513 PMC7343571

[B6] Fliedner A. Kirchner P. Wiesener A. van de Beek I. Waisfisz Q. van Haelst M. (2020). Variants in SCAF4 cause a neurodevelopmental disorder and are associated with impaired mRNA processing. Am. J. Hum. Genet. 107 (3), 544–554. 10.1016/j.ajhg.2020.06.019 32730804 PMC7477272

[B7] Fujimoto S. Takase T. Kadono N. Maekubo K. Hirai Y. (2014). Krtap11-1, a hair keratin-associated protein, as a possible crucial element for the physical properties of hair shafts. J. Dermatol. Sci. 74 (1), 39–47. 10.1016/j.jdermsci.2013.12.006 24439038

[B8] Gregersen L. H. Mitter R. Ugalde A. P. Nojima T. Proudfoot N. J. Agami R. (2019). SCAF4 and SCAF8, mRNA anti-terminator proteins. Cell 177 (7), 1797–1813. 10.1016/j.cell.2019.04.038 31104839 PMC6579486

[B9] Hanenberg E. H. A. T. Knol E. F. Merks J. W. M. (2001). Estimates of genetic parameters for reproduction traits at different parities in Dutch Landrace pigs. Livest. Prod. Sci. 69 (2), 179–186. 10.1016/s0301-6226(00)00258-x

[B10] Hidalgo A. M. Lopes M. S. Harlizius B. Bastiaansen J. W. M. (2016). Genome-wide association study reveals regions associated with gestation length in two pig populations. Anim. Genet. 47 (2), 223–226. 10.1111/age.12395 26667091

[B11] Hollema B. L. Zwiers S. Hermesch S. (2020). Genetic parameters for haemoglobin levels in sows and piglets as well as sow reproductive performance and piglet survival. Animal 14 (4), 688–696. 10.1017/S1751731119002532 31657286

[B12] Huang L. Tang X. Shi X. Su L. (2020). miR-532-5p promotes breast cancer proliferation and migration by targeting RERG. Exp. Ther. Med. 19 (1), 400–408. 10.3892/etm.2019.8186 31853317 PMC6909632

[B13] Imboonte N. Kuhaaudomlarp P. (2012). Genetic associations between stillbirth, total number of piglets born and gestation length in a commercial pig farm. Thai J. Vet. Med. 42 (2), 165–172.

[B14] Knutson D. C. Clagett-Dame M. (2015). A complex RARE is required for the majority of Nedd9 embryonic expression. Transgenic Res. 24 (1), 123–134. 10.1007/s11248-014-9825-9 25120220 PMC4274375

[B15] Kober F. X. Koelmel W. Kuper J. Drechsler J. Mais C. Hermanns H. M. (2013). The crystal structure of the protein-disulfide isomerase family member ERp27 provides insights into its substrate binding capabilities. J. Biol. Chem. 288 (3), 2029–2039. 10.1074/jbc.M112.410522 23192347 PMC3548509

[B16] Leigh A. O. (1981). Factors affecting the gestation period of pigs in Nigeria. Trop. Anim. Health Prod. 13 (2), 87–93. 10.1007/BF02237898 7233562

[B17] Li M. Han Y. Zhou H. Li X. Lin C. Zhang E. (2018). Transmembrane protein 170B is a novel breast tumorigenesis suppressor gene that inhibits the Wnt/β-catenin pathway. Cell Death Dis. 9 (2), 91. 10.1038/s41419-017-0128-y 29367600 PMC5833782

[B18] Luo C. Pook E. Wang F. Archacki S. R. Tang B. Zhang W. (2020). ADTRP regulates TFPI expression via transcription factor POU1F1 involved in coronary artery disease. Gene 753, 144805. 10.1016/j.gene.2020.144805 32445923

[B19] Lupu C. Patel M. M. Lupu F. (2021). Insights into the functional role of ADTRP (Androgen-Dependent TFPI-regulating protein) in health and disease. Int. J. Mol. Sci. 22 (9), 4451. 10.3390/ijms22094451 33923232 PMC8123165

[B20] Ma H-L. Gong F. Tang Y. Li X. Li X. Yang X. (2015). Inhibition of endometrial tiam1/rac1 signals induced by miR-22 up-regulation leads to the failure of embryo implantation during the implantation window in pregnant mice. Biol. Reprod. 92 (6), 152. 10.1095/biolreprod.115.128603 25926441

[B21] Nagy I. Curik I. Farkas J. Csato L. Csornyei Z. (2009). Bayesian inference of genetic parameters on litter size and gestation length in Hungarian Landrace and Hungarian Large White pigs. Ital. J. Anim. Sci. 8, 68–70. 10.4081/ijas.2009.s3.68

[B22] Ogawa S. Konta A. Kimata M. Ishii K. Uemoto Y. Satoh M. J. A. S. J. (2019). Estimation of genetic parameters for farrowing traits in purebred Landrace and Large White pigs. Animal Sci. J. 90 (1), 23–28. 10.1111/asj.13120 PMC6587850 30370591

[B23] Omtvedt I. T. Stanislaw C. M. Whatley J. A. Jr. (1965). Relationship of gestation length, age and weight at breeding, and gestation gain to sow productivity at farrowing. J. Anim. Sci. 24 (2), 531–535. 10.2527/jas1965.242531x 14324380

[B24] Onteru S. K. Fan B. Du Z. Q. Garrick D. J. Stalder K. J. Rothschild M. F. (2012). A whole-genome association study for pig reproductive traits. Anim. Genet. 43 (1), 18–26. 10.1111/j.1365-2052.2011.02213.x 22221021

[B25] Rizzo H. E. Escaname E. N. Alana N. B. Lavender E. Gelfond J. Fernandez R. (2020). Maternal diabetes and obesity influence the fetal epigenome in a largely Hispanic population. Clin. Epigenetics 12 (1), 34. 10.1186/s13148-020-0824-9 32075680 PMC7031937

[B26] Rydhmer L. Lundeheim N. Canario L. (2008). Genetic correlations between gestation length, piglet survival and early growth. Livest. Sci. 115, 287–293. 10.1016/j.livsci.2007.08.014

[B27] Sasaki M. M. Nichols J. T. Kimmel C. B. (2013). edn1 and hand2 Interact in early regulation of pharyngeal arch outgrowth during zebrafish development. PLoS One 8 (6), e67522. 10.1371/journal.pone.0067522 23826316 PMC3691169

[B28] See G. M. Trenhaile-Grannemann M. D. Spangler M. L. Ciobanu D. C. Mote B. E. (2019). A genome-wide association study for gestation length in swine. Anim. Genet. 50 (5), 539–542. 10.1111/age.12822 31297858

[B29] Su D. Kosciuk T. Yang M. Price I. R. Lin H. (2021). Binding affinity determines substrate specificity and enables discovery of substrates for N-myristoyltransferases. ACS Catal. 11 (24), 14877–14883. 10.1021/acscatal.1c03330 34956690 PMC8689648

[B30] Thaete L. G. Jilling T. Synowiec S. Khan S. Neerhof M. G. (2007). Expression of endothelin 1 and its receptors in the hypoxic pregnant rat. Biol. Reprod. 77 (3), 526–532. 10.1095/biolreprod.107.061820 17554077 PMC1989130

[B31] Ullrey D. E. Sprague J. I. Becker D. E. Miller E. R. (1965). Growth of the swine fetus. J. Anim. Sci. 24, 711–717. 10.2527/jas1965.243711x 14313732

[B32] van den Berg S. Vandenplas J. van Eeuwijk F. A. Bouwman A. C. Lopes M. S. Veerkamp R. F. (2019). Imputation to whole-genome sequence using multiple pig populations and its use in genome-wide association studies. Genet. Sel. Evol. 51, 2. 10.1186/s12711-019-0445-y 30678638 PMC6346588

[B33] Wang X. Bi X. Huang X. Wang B. Guo Q. Wu Z. (2020). Systematic investigation of biomarker-like role of ARHGDIB in breast cancer. Cancer Biomark. 28 (1), 101–110. 10.3233/CBM-190562 32176626 PMC12662329

[B34] Wang Y. Ding X. Tan Z. Xing K. Yang T. Pan Y. (2018). Genome-wide association study for reproductive traits in a Large White pig population. Anim. Genet. 49 (2), 127–131. 10.1111/age.12638 29411893 PMC5873431

[B35] Wang Z. Zhang Z. Chen Z. Sun J. Cao C. Wu F. (2022). Author correction: Pharp: A pig haplotype reference panel for genotype imputation. Sci. Rep. 12 (1), 13964. 10.1038/s41598-022-18078-y 35977973 PMC9385863

[B36] Wilkie P. J. Paszek A. A. Beattie C. W. Alexander L. J. Wheeler M. B. Schook L. B. (1999). A genomic scan of porcine reproductive traits reveals possible quantitative trait loci (QTLs) for number of corpora lutea. Mamm. Genome 10 (6), 573–578. 10.1007/s003359901047 10341088

[B37] Williams C. B. Phelps-Polirer K. Dingle I. P. Williams C. J. Rhett M. J. Eblen S. T. (2020). HUNK phosphorylates EGFR to regulate breast cancer metastasis. Oncogene 39 (5), 1112–1124. 10.1038/s41388-019-1046-5 31597954 PMC6989402

[B38] Yang A. Q. Chen B. Ran M. L. Yang G. M. Zeng C. (2020). The application of genomic selection in pig cross breeding. Yi Chuan 42 (2), 145–152. 10.16288/j.yczz.19-253 32102771

[B39] Yue Y. Zhang C. Zhao X. Liu S. Lv X. Zhang S. (2021). Tiam1 mediates Rac1 activation and contraction-induced glucose uptake in skeletal muscle cells. Faseb J. 35 (2), e21210. 10.1096/fj.202001312R 33225507

[B40] Zhang Y. Zhao W. Xu H. Hu M. Guo X. Jia W. (2019). Hyperandrogenism and insulin resistance-induced fetal loss: Evidence for placental mitochondrial abnormalities and elevated reactive oxygen species production in pregnant rats that mimic the clinical features of polycystic ovary syndrome. J. Physiol. 597 (15), 3927–3950. 10.1113/JP277879 31206177

