# Supplementary figures and images for: Genetic parameters estimation and genome molecular marker identification for gestation length in pigs
Source: Front Genet. 2023 Jan 5;13:1046423. doi: 10.3389/fgene.2022.1046423 (PMC9849246; doi:10.3389/fgene.2022.1046423)

A

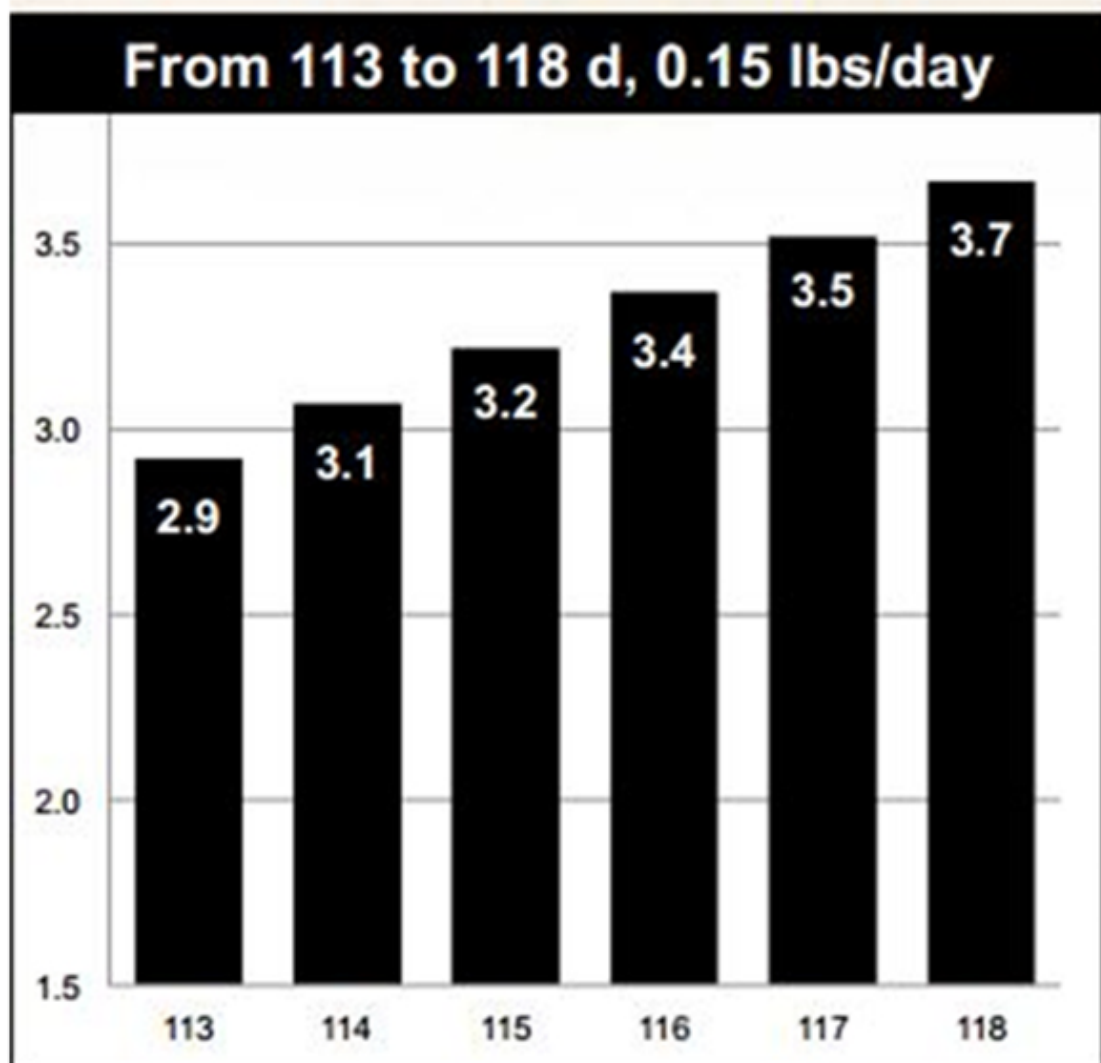

B

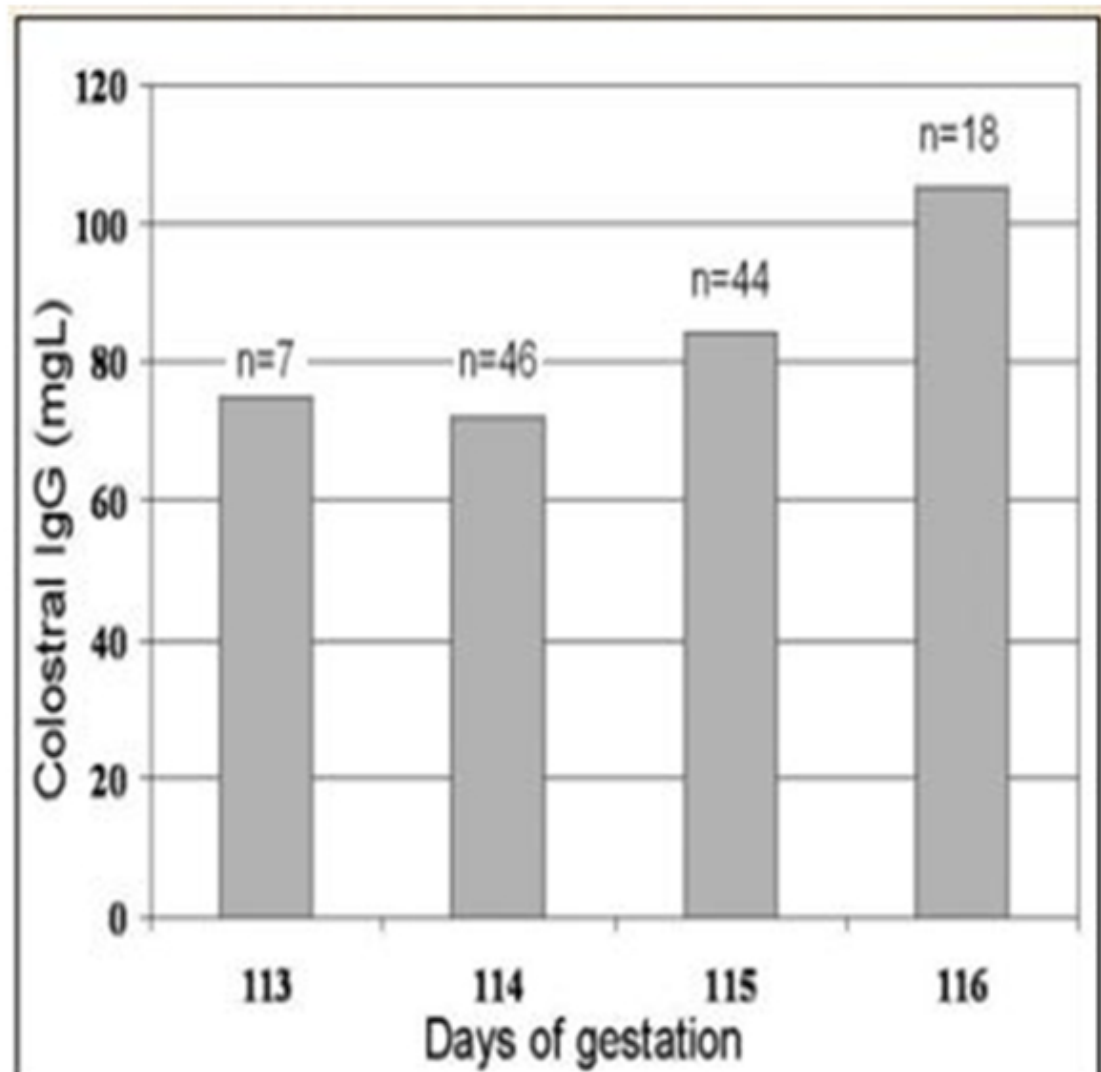

Supplement: Supplementary file 4 [file Image1.pdf]
